# The role of personality in posttraumatic stress disorder, trait resilience, and quality of life in people exposed to the Kiss nightclub fire

**DOI:** 10.1371/journal.pone.0220472

**Published:** 2019-07-29

**Authors:** Vitor Crestani Calegaro, Pedro Henrique Canova Mosele, Bianca Lorenzi Negretto, Cleonice Zatti, Angelo Batista Miralha da Cunha, Lucia Helena Machado Freitas

**Affiliations:** 1 Department of Neuropsychiatry, Federal University of Santa Maria (UFSM), Santa Maria, Brazil; 2 Doctoral Program in Psychiatry and Behavioral Sciences, Federal University of Rio Grande do Sul (UFRGS), Porto Alegre, Brazil; 3 Medical Residency in Psychiatry, UFSM, Santa Maria, Brazil; 4 Course of Medicine, UFSM, Santa Maria, Brazil; 5 Department of Psychiatry and Legal Medicine, UFRGS, Porto Alegre, Brazil; Technion Israel Institute of Technology, ISRAEL

## Abstract

**Objective:**

To evaluate the relationship among personality (according to Cloninger’s psychobiological model), posttraumatic stress disorder (PTSD) symptoms, trait resilience and quality of life (QoL) in people who were exposed to the Kiss nightclub fire.

**Methods:**

188 participants were assessed with the Posttraumatic Checklist–civilian version (PCL-C), the Resilience Scale (RS), the Temperament and Character Inventory (TCI), the World Health Organization Quality of Life–Bref (WHOQOL-Bref), and the WHOQOL-100 Spirituality, religiousness, and personal beliefs (WHOQOL-100-SRPB). Data were analyzed in a dimensional approach, with correlation analysis, multiple linear regression and Structural Equation Modeling (SEM), with PCL-C, RS, and WHOQOL-Bref dimensions as dependent variables.

**Results:**

Multiple linear regression showed that PTSD symptoms were predicted by harm avoidance (β = .34, p < .001), self-directedness (β = -.28, p < .01), and self-transcendence (β = .24, p < .01). Trait resilience was predicted by harm avoidance (β = -.38, p < .01), self-directedness (β = .20, p < .05), and self-transcendence (β = .18, p < .05). Also, PTSD symptoms had considerable negative effect on all dimensions of QoL. Self-transcendence was a positive predictor of subjective and spiritual QoL. SEM showed that QoL was predicted by PTSD symptoms (β = -.52, p < .001), trait resilience (β = .30, p < .001), cooperativeness (β = .135, p = 0.40), and self-directedness (β = .27, p < .01). The effect of self-directedness on QoL was mediated by PTSD symptoms and trait resilience. PTSD symptoms also mediated the relationship between trait resilience and QoL, and RS mediated the relationship of personality and PTSD symptoms.

**Conclusion:**

The study gives insights on prediction of PTSD severity, trait resilience and QoL from temperament and character traits, in a sample of people exposed to the Kiss nightclub fire. Harm avoidance was the most influent trait on PTSD symptoms and trait resilience. Self-directedness was the most import trait related to QoL, still that it was more related to PTSD severity than personality traits. Self-transcendence had positive effects on both PTSD symptoms and trait resilience, indicating a coping style that may coexist with psychopathology.

## Introduction

On January 27, 2013, a deadly fire deeply wounded the city of Santa Maria, located in the south of Brazil. The ignition of an improper pyrotechnic artifact during a show in a crowded nightclub resulted in the death of 242 people and left more than 600 injured [1–3]. One to two years after disasters like this, about 25–75% of survivors and 5–40% of first responders may be diagnosed with posttraumatic stress disorder (PTSD), a mental disorder that can be chronic and disabling [4,5]. Moreover, the quality of life (QoL) of such individuals is inversely related to the severity of their PTSD symptoms—that is, the more symptomatic, the lower the QoL [6–9]. On the other hand, a substantial proportion of people exposed to trauma do not develop mental disorders. Such individuals may be considered resilient, adapting relatively well under severe stress.

The construct of resilience has slight differences between authors, but they agree that it describes the ability to successfully function in the face of adversity [10]. The capacity to cope with fears, experience positive emotions, reformulate stressful events, and feel supported by social relations is based on the more adaptive functioning of circuits of fear, reward, emotional regulation, and social behavior [11]. In this decade, the related epigenetic mechanisms have been described based on the interaction of genetic and biological predispositions with life events as a person develops, which results in individual characteristics that make people more or less vulnerable to stress [12]. In other words, adaptive personality traits developed in this process might be protective factors, whereas maladaptive traits might be risk factors for mental disorders [13].

There is a discussion on whether resilience is a personality trait or state. Most studies measure resilience as the presence or absence of psychopathology, while another approach is to measure resilience as the individual’s perceived ability to cope with adversities [14]. The former approach is based on low PTSD symptoms (state) and the latter on assessment scales (trait). Studies that directly measured resilience using a validated instrument found an association with high extraversion, high agreeableness, low neuroticism [15–17], high positive emotionality and low negative emotionality [18,19], low harm avoidance and high persistence, self-directedness, and cooperativeness [20–22]. Rutter (2007) argued that resilience cannot be a personality trait, as people may be resilient to some hazards but not others, and to some outcomes but not others; moreover, a person will only be resilient in the face of adversity, trauma, or stress [23]. Different concepts of resilience gave rise to a variety of techniques and scales to measure it. Scoloveno (2017) reviewed this topic and concluded that the Resilience Scale (RS) developed by Wagnild and Young (1993) is the only instrument that conceptualizes resilience as a personality characteristic (trait resilience) that has been used across different age groups [10,24].

Studies that assessed PTSD severity (thus, low resilience) found that the symptoms are positively related to negative emotionality, neuroticism, harm avoidance, novelty seeking, self-transcendence, hostility/anger, and trait anxiety; and negatively with extraversion, conscientiousness, self-directedness, the combination of high positive and low negative emotionality, hardiness, and optimism [25]. The assessment of personality in traumatized people may contribute to identifying individuals at risk of developing PTSD, planning interventions based on personality dysfunction, and predicting treatment responses [26].

Of the personality models, the Psychobiological Model of Temperament and Character is the most congruent with the current epigenetic perspective of behavioral development [13]. In this model, personality is composed of two interacting structures: temperament and character [27]. Temperament is innate, heritable, stable over time, conditioned by procedural learning, and refers to skills and habits elicited by automatic associative responses to simple emotional stimuli. It is comprised of four traits: harm avoidance (the trend toward behavioral inhibition and to respond intensely to aversive stimuli), novelty seeking (the bias to initiate behaviors in response to novelty), reward dependence (a tendency to respond intensely to social approval), and persistence (resistance to interrupting the behavior even when confronted by frustration or the absence of a reward). Character is more malleable, involving high cognitive functions and propositional learning; it refers to ego strengths, maturity, personal objectives, and values, and reflects different aspects of self-object relations. It is composed of three traits: self-directedness (the perception of autonomy and resources to achieve personal goals), cooperativeness (that reflects empathetic identification and tolerance through others), and self-transcendence (self-awareness of being an integral part of the universe, altruism, and spiritual acceptance) [27–29].

Despite there being evidence that temperament and character have a relationship with trait resilience, studies have not assessed the presence of exposure to trauma and psychopathology [20–22]. Additionally, in other studies, temperament and character traits have been related to PTSD; but in these cases, state resilience was considered as the absence of the disorder [30,31]. A broader examination of resilience, including the notions of both trait and state, its relationship to personality traits, and how it manifests in functioning, may add to the comprehension of individual characteristics related to healthy responses to trauma. In this line, the Psychobiological Model of Temperament and Character has an advantage in relation to other personality models: it does not only measure personal characteristics, but also personality functioning, which may be easily translated to the clinical practice [13]. The aim of this study was to investigate the role of personality in predicting PTSD severity, trait resilience, and QoL in people exposed to the Kiss nightclub fire. We hypothesized that (1) PTSD severity and trait resilience are both predicted by personality traits; (2) QoL is predicted by PTSD severity, trait resilience, and personality traits; and (3) PTSD severity and trait resilience mediate the effect of personality traits on QoL.

## Methods

### Participants

The participants were 188 people directly exposed to the event as victims (n = 120; 63.8%) or first responders (n = 68; 36.2%), assessed in the Integrated Care Center for Accident Victims, a multi-professional center located at the University Hospital of Santa Maria [32]. They were interviewed by the research team after consultation in the respiratory and psychiatric clinics between January 2015 and December 2017. All participants gave informed consent. The sample characteristics are shown in Table 1. Of the victims, 111 (92.5%) were survivors and 9 (7.5%) witnessed the event. Of the first responders, 42 (61.8%) were military police officers, 23 (33.8%) were firefighters, 2 (1.7%) were military (1.7%), and 1 (1.5%) was a civil police officer. Most of the individuals had inhaled smoke during the fire (n = 169; 89.9%). Only a quarter of the participants (n = 47; 25.1%) declared having a psychiatric disorder prior to the disaster, and 40 (21.3%) had undergone prior psychiatric treatment.

**Table 1:** Sample characteristics and comparison between type of exposure.

	Victims (N = 120)		First responders (N = 68)		Total (N = 188)		
	N	%	N	%	N	%	p
Male sex	55	45.8%	58	85.3%	113	60.1%	< .001
Age (median; min-max)	25	20–56	34	24–57	18	20–57	.007
Years of study (median; min-max)	14	2–24	13	6–19	13	1–24	< .001
Ethnicity							
White	106	88.3%	57	85.1%	163	87.2%	.523
Non-white	14	11.7%	10	14.9%	24	12.8%	
Marital status							
Single	94	78.3%	13	19.2%	107	56.9%	< .001
Married	20	16.7%	46	67.6%	66	35.1%	
Divorced/widowed	6	5.0%	9	13.2%	15	8.0%	
Occupation							
Employed	56	46.7%	65	95.6%	121	64.4%	< .001
Unemployed	18	15.0%	0	0.0%	18	9.6%	
Retired/sick leave	7	5.8%	3	4.4%	10	5.3%	
Student	39	32.5%	0	0.0%	39	20.7%	
Current psychiatric treatment	69	57.5%	16	23.9%	85	45.5%	<0,001
Type of treatment							
No treatment	58	48.3%	52	77.6%	110	58.8%	< .001
Psychopharmaceutical only	20	16.7%	4	6.0%	24	12.8%	
Psychoterapy only	14	11.7%	8	11.9%	22	11.8%	
Psychopharmaceutical plus psychotherapy	28	23.3%	3	4.5%	31	16.6%	

P-values for Chi-square test (categorical) and Mann-Whitney test (continuous). There was one missing for ethnicity and two for marital status.

### Measures

Individuals were assessed with the following instruments: the Posttraumatic Checklist—civilian version (PCL-C), the Temperament and Character Inventory (TCI), the RS, and the World Health Organization Quality of Life–Bref (WHOQOL-Bref).

The PCL-C is a 17-question self-report questionnaire used for screening and monitoring PTSD [33]. The Portuguese version has good internal consistency, test-retest reliability, and factorial validity [34,35]. Although it is based on the DSM-IV, its measures have sensitivity and specificity similar to instruments developed using the DSM-5 and PCL-5 [36].

The TCI is a self-report instrument developed to assess personality according to Cloninger’s Psychobiological Model of Temperament and Character [27]. This version has 240 true-false questions, translated into Brazilian Portuguese and validated [37]. Each trait is defined as a bipolar continuum, from low to high scores, capturing normal and extreme presentations. Domains are independent and composed of different facets.

The RS measures levels of positive psychosocial adaptation to important life events, and was used for evaluation of trait resilience [24]. It uses 25 items in the form of Likert scales; each item has a score from 1 (totally disagree) to 7 (totally agree). Scores range from 25 to 175, with higher scores meaning higher resilience. The Portuguese translated scale has shown high internal consistency and construct and discriminant validity [38].

The WHOQOL-Bref is a self-report instrument developed by the World Health Organization to measure QoL; it has been translated into Portuguese [39]. It has 26 questions—2 assessing general QoL (Q1: general QoL; Q2: health-related QoL) and the other 24 divided into 4 domains: physical, psychological, social relations, and environment. Spirituality, religiousness, and personal beliefs (SRPB) were assessed using the respective domain of the WHOQOL-100. It comprises four questions that ask about the meaning of life and personal beliefs. Despite the SRPB domain being considered insufficient to encompass the complexity of the construct of religiousness/spirituality, it has good internal consistency and convergent validity with the complete WHOQOL-SRPB, thus this scale was used as an approximated measure of spiritual QoL [40].

### Data analysis

The data were analyzed using Statistical Package for the Social Sciences (SPSS), 23th version, and MPlus, 7^th^ version. The univariate analysis was done first, and outliers were corrected. Normality was assessed using skewness, kurtosis, histograms, and normality tests. The data showed a slight deviance from normality; thus, we performed parametric tests with bootstrapping for bias corrected and accelerated 95% confidence intervals (Bca 95% CI), using 2,000 samples. Since victims and first responders (type of exposure) might be considered as subsamples, which might influence the measures, as well as sex differences and psychiatric treatment, analyses were controlled by these variables. Thus, partial correlations between the variables PCL-C, RS, the TCI dimensions, the WHOQOL-Bref dimensions, and SRPB were employed, controlling for sex, type of exposure, and current psychiatric treatment. All tests used a level of significance of .05.

The multivariable analyses included hierarchical multiple linear regression using PCL-C; RS; Q1; Q2; physical, psychological, social, and environmental WHOQOL-Bref dimensions; and SRPB as dependent variables (models 1 to 9). Data were included in three steps, using a forced entry procedure. In the first step, we entered the variables sex, type of exposure, and current psychiatric treatment, for control purpose. The second step included the RS and PCL-C (in models 1 and 2, only one of them was entered). The third step added the temperament (HA, P, NS, RD) and character dimensions (SD, CO, ST). Again, the models had confidence intervals and significance tests bootstrapped because this method does not rely on assumptions of normality and homoscedasticity.

Finally, in order to examine the complex relationships among the variables, structural equation modeling (SEM) was performed, combining confirmatory factor analysis (CFA), multiple linear regression, and path analyses. CFA was used to model the latent variable QOL from the WHOQOL dimensions. Residuals of factor measures were set as uncorrelated. Then, multiple linear regression used QOL as the main outcome, temperament and character traits as the predictors, and PCL-C and the RS as mediators. RS was tested as a predictor of PCL-C. SRPB was regressed to self-transcendence, as they are constructs directly related. Type of exposure, sex, and current psychiatric treatment were included as controlling covariates in all multiple linear regressions. Estimator was maximum likelihood with standard errors robust to non-normality and non-independence. Coefficients were standardized to allow comparisons. We tested diverse models, using different paths and relationships among the variables. Models were compared using goodness of fit measures (GoF): root mean square error of approximation (RMSEA < .08, with p ≥ .05), CFI and TLI ≥ .95, and standardized root mean square residual (SRMR < .08) [41]. We present in the results only the best fitting model. Models that included the variables Q1 and Q2 presented worse GoF measures.

## Results

Considering that sex, type of exposure, and current treatment are factors that may influence real correlations among personality and outcomes, we performed partial correlations controlling for these variables (Table 2). The variables that remained statistically significant for the bootstrapped confidence intervals were the same. PTSD symptoms had a moderate and negative correlation with trait resilience, and both variables showed opposite correlations with the same variables, except for self-transcendence, which was not correlated with trait resilience. Regarding personality, PTSD symptoms were positively correlated with harm avoidance and self-transcendence, and negatively with self-directedness and cooperativeness. Moderate to strong correlations were found between PTSD symptoms and trait resilience, with all aspects of QoL. Self-directedness was strongly and negatively correlated with harm avoidance, and strongly and positively correlated with cooperativeness (see discussion below). Self-directedness and cooperativeness were correlated with other personality traits, excepting novelty seeking and self-transcendence. Harm avoidance, self-directedness, and cooperativeness were also correlated with all aspects of QoL, while self-transcendence showed a moderate correlation with spiritual QoL.

**Table 2:** Partial correlations controlled for sex, exposure, and current treatment, with bias corrected and accelerated 95% confidence intervals (Bca 95% CI), based on 2,000 samples.

	PCL-C	RS	NS	HA	RD	P	SD	C	ST	Q1	Q2	PHYS	PSYCH	SOCIAL	ENVIR	SRPB
**PCL-C**	1.00	-.60, -.34	-.27, .02	.48, .74	-.33, .01	-.30, .06	-.69, -.47	-.48, -.16	.06, .36	-.58, -.30	-.54, -.25	-.74, -.50	-.77, -.53	-.61, -.35	-.58, -.34	-.52, -.21
**RS**	-.48***	1.00	-.13, .20	-.66, -.42	-.10, .31	.01, .36	.33, .62	.05, .38	-.11, .27	.15, .49	.15, .48	.41, .67	.50, .73	.24, .56	.19, .48	.20, .56
**NS**	-.13	.04	1.00	-.42, -.11	-.05, .31	-.25, .10	-.24, .07	-.25, .09	.05, .35	-.11, .25	-.15, .24	-.08, .23	-.08, .25	-.05, .28	-.23, .13	.03, .36
**HA**	.62***	-.55***	-.27**	1.00	-.35, .00	-.40, -.06	-.70, -.51	-.43, -.14	-.24, .13	-.40, -.08	-.44, -.11	-.67, -.40	-.73, -.50	-.54, -.24	-.51, -.24	-.50, -.22
**RD**	-.16	.11	.14	-.18*	1.00	-.08, .29	.03, .40	.38, .65	-.09, .24	-.06, .35	-.01, .32	.02, .39	-.05, .35	.10, .47	-.08, .26	.00, .37
**P**	-.12	.18*	-.07	-.23**	.11	1.00	.05, .38	.07, .41	-.08, .27	.00, .39	-.11, .24	-.05, .34	-.02, .38	.05, .42	.00, .36	-.07, .34
**SD**	-.58***	.48***	-.09	-.61***	.22*	.22*	1.00	.39, .66	-.31, .02	.19, .49	.17, .49	.45, .70	.53, .75	.31, .60	.35, .65	.18, .53
**C**	-.32***	.22*	-.07	-.29**	.53***	.25**	.53***	1.00	-.29, .08	.04, .39	.05, .39	.16, .48	.20, .50	.21, .54	.08, .43	.01, .40
**ST**	.21*	.08	.20*	-.05	.08	.09	-.15	-.09	1.00	-.07, .29	-.19, .20	-.16, .16	-.16, .19	-.17, .15	-.26, .09	.13, .47
**Q1**	-.45***	.31***	.08	-.24**	.16	.20*	.35***	.22*	.11	1.00	.32, .60	.47, .68	.50, .72	.43, .70	.38, .65	.38, .64
**Q2**	-.40***	.33***	.05	-.28**	.16	.06	.33***	.22*	.00	.47***	1.00	.50, .71	.39, .66	.26, .55	.22, .51	.14, .45
**PHYS**	-.63***	.55***	.08	-.54***	.22*	.15	.58***	.32***	-.01	.58***	.61***	1.00	.76, .89	.46, .68	.39, .65	.30, .60
**PSYCH**	-.66***	.62***	.09	-.62***	.17	.19*	.65***	.35***	.01	.62***	.53***	.83***	1.00	.58, .78	.48, .69	.27, .62
**SOCIAL**	-.49***	.41***	.12	-.39***	.30**	.25**	.46***	.38***	-.02	.58***	.42***	.58***	.69***	1.00	.42, .66	.19, .50
**ENVIR**	-.47***	.34***	-.06	-.38***	.09	.18	.50***	.26**	-.09	.53***	.38***	.53***	.59***	.54***	1.00	.22, .53
**SRPB**	-.37***	.38***	.20*	-.36***	.20*	.14	.36***	.22*	.32***	.52***	.30***	.46***	.45***	.35***	.38***	1.00

* p < .05

** p < .01

*** p < .001.

Low table: Pearson’s *r* coefficient. Upper table: Bca 95% CI for *r*. Bold values are significant.

PCL-C: Posttraumatic checklist—civilian version; RS: Resilience Scale; NS: novelty seeking; HA: harm avoidance; RD: reward dependence; P: persistence; SD: self-directedness; C: cooperativeness; ST: self-transcendence. Q1: general quality of life (QoL); Q2: health-related QoL; PHYS: physical QoL; PSYCH: psychological QoL; ENVIR: environment QoL; SRPB: spirituality, religiousness, personal beliefs (spiritual QoL).

The results of the multiple linear regression for the outcomes are shown in Table 3. All models were significant, although the third step did not show significant change (ΔF) in relation to the second for health-related QoL, physical QoL, and environmental QoL. In the second step, PTSD symptoms and trait resilience were both significant predictors of one another. Furthermore, in the third step, when temperament and character were taken into account, PTSD symptoms were not found to be a significant predictor of trait resilience, and vice-versa; thus, variances in both measures were better explained by personality. Notably, harm avoidance and self-directedness had opposite effects on PTSD symptoms and trait resilience, but self-transcendence was a positive predictor of both traits.

**Table 3:** Standardized coefficients (β) for hierarchical multiple linear regression models for PCL-C, RS, and WHOQOL dimensions as outcomes, using RS, PCL-C, and personality traits as predictors, controlling for sex, exposure, and current psychiatric treatment.

	PCL-C	RS	Q1	Q2	PHYS	PSYCH	SOCIAL	ENVIR	SRPB
**Step 1**									
***F***	11.21***	5.30**	9.05***	0.19***	11.29***	11.73***	3.65*	3.46*	5.15**
***R***^***2***^	.16	.08	.18	.19	.21	.22	.08	.08	.11
**Sex**	.01	-.19*	-.14	-.02	-.22*	-.20	-.04	-.24*	-.17
**Exposure**	.00	.08	-.13	.36**	.00	.09	.05	-.02	.08
**Current treatment**	.42***	-.11	-.34**	-.12	-.32**	-.29**	-.24*	-.09	-.16
**Step 2**									
***ΔF***	27.03***	27.03***	15.04***	16.07***	56.64***	73.51***	23.26***	17.34***	13.52***
***ΔR***^***2***^	.12	.14	.16	.17	.38	.42	.25	.20	.16
**Sex**	-.06	-.19*	.04	.04	-.14	-.11	.02	-.19	-.12
**Exposure**	.03	.08	.16	.37**	-.01	.07	.04	-.01	.07
**Current treatment**	.38***	.06	-.17	.03	-.08	-.05	-.04	.10	-.02
**PCL-C**	-	-.41***	-.40***	-.34**	-.46***	-.46***	-.41***	-.41***	-.25*
**RS**	-.37***	-	.07	.17*	.32***	.37***	.23*	.15	.26*
**Step 3**									
***ΔF***	13.77***	6.18***	2.53*	0.53	1.69	3.14**	2.51*	2.00	4.41***
***ΔR***^***2***^	.28	.18	.09	.02	.04	.06	.09	.08	.15
**Sex**	-.09	.01	-0.3	-.02	-.17	-.07	-.05	-.13	-.13
**Exposure**	.06	-.03	.16	.38***	-.03	.04	.04	-.04	.03
**Current treatment**	.28***	-.02	-.19*	.03	-.12	-.11	-.09	.06	-.09
**PCL-C**	-	-.14	-.48***	-.36**	-.38***	-.32**	-.30*	-.24*	-.26*
**RS**	-.10	-	.02	.17	.25	.25**	.17	.04	.14
**NS**	-.09	-.08	.06	.02	.02	.03	.11	-.05	.13
**HA**	.34***	-.38**	.29*	.17	.05	-.06	.10	-.04	.11
**RD**	-.04	.03	.05	.06	.09	-.01	.12	-.02	.05
**P**	.03	.04	.13	-.02	.00	.01	.13	.05	.01
**SD**	-.28**	.20*	.22	.11	.23*	.27**	.18	.34**	.25
**C**	-.01	-.03	-0.2	.02	-.02	.02	.10	-.02	.01
**ST**	.24**	.18*	.23**	.06	.07	.09	.03	.02	.35**

β means how many standard deviations the outcome variable will change, per standard deviation increase in the predictor variable, when all other variables remain constant.

PCL-C: Posttraumatic checklist—civilian version; RS: Resilience Scale; NS: novelty seeking; HA: harm avoidance; RD: reward dependence; P: persistence; SD: self-directedness; C: cooperativeness; ST: self-transcendence. Q1: general quality of life (QoL); Q2: health-related QoL; PHYS: physical QoL; PSYCH: psychological QoL; SOCIAL: social QoL; ENVIR: environment QoL; SRPB: spirituality, religiousness, personal beliefs (spiritual QoL).

* p < .05

** p < .01

***p < .001. All p-values correspond to the 95% bias corrected and accelerated confident intervals. Bold values are statistically significant.

PTSD symptoms had a considerable negative effect on all dimensions of QoL, even considering the personality traits. On the other hand, when taking into account temperament and character, psychological QoL was independently predicted by trait resilience and self-directedness. Although self-directedness had been a significant predictor of physical and environmental QoL, the inclusion of personality traits did not add significant information to the model (ΔF). Finally, spiritual QoL was better predicted by PTSD symptoms and self-transcendence (third step) than trait resilience and PTSD symptoms (second step).

**Fig 1:**
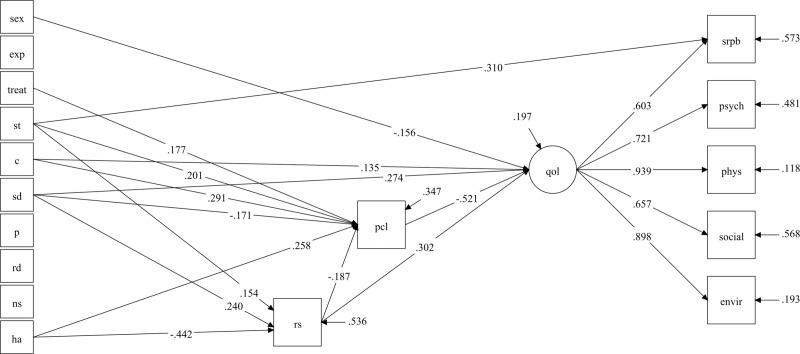
Structural equation model using the latent variable QOL, composed of the WHOQOL-Bref dimensions, as the main outcome. Notes: Standardized coefficients are displayed only for significant relationships. Treat: current psychiatric treatment; exp: type of exposure; pcl: Posttraumatic checklist—civilian version; rs: Resilience Scale; ns: novelty seeking; ha: harm avoidance; rd: reward dependence; p: persistence; sd: self-directedness; c: cooperativeness; st: self-transcendence; srpb: spiritual QoL; phys: physical QoL; psych: psychological QoL; social: social QoL; envir: environment QoL.

Finally, the results of the SEM are shown in Fig 1. The latent variable QOL was composed of the physical, psychological, social, and environmental dimensions of the WHOQOL-Bref, and of spiritual QoL. The model resulted in adequate GoF indices: $X_{52}^{2}=52.903$, p = .439; RMSEA = .010 [.000; .051], p = .944; CLI = .999; TLI = .998; SRMR = .027. The explained variance (*R*^*2*^) ranged from .43 (SRPB) to .88 (WHOQOL-Physical). Self-directedness had the higher total effect in the model and was the only trait that had a direct effect on QoL. Indirect effects, p-values, and confidence intervals are shown in Table 4.

**Table 4:** SEM standardized coefficients with 95% confidence intervals (CI) for indirect effects, and p-values.

		95% CI				
	Coefficient	Lower	Upper	S.E.	Coef./S.E.	p
**Indirect effect of RS on QOL via PCL-C**	**.097**	**.013**	**.182**	**.043**	**2.257**	**.024**
**Total indirect effect of SD on QOL**	**.113**	**.029**	**.196**	**.043**	**2.645**	**.008**
**SD→PCL-C→QOL**	**.089**	**.009**	**.17**	**.041**	**2.176**	**.030**
SD**→**RS**→**PCL-C**→**QOL	.023	-.001	.047	.012	1.909	.056
**Indirect effect of C on QOL via PCL-C**	**-.152**	**-.228**	**-.076**	**.039**	**-3.931**	**< .001**
**Total indirect effect of ST on QOL**	**-.090**	**-.155**	**-.025**	**.033**	**-2.703**	**.007**
**ST→PCL-C→QOL**	**-.105**	**-.173**	**-.037**	**.035**	**-3.019**	**.003**
ST**→**RS**→**PCL-C**→**QOL	.015	-.002	.032	.009	1.723	.085
**Total indirect effect of HA on QOL**	**-.177**	**-.276**	**-.079**	**.05**	**-3.526**	**< .001**
**HA→PCL-C→QOL**	**-.134**	**-.228**	**-.041**	**.048**	**-2.827**	**.005**
**HA→RS→PCL-C→QOL**	**-.043**	**-.084**	**-.002**	**.021**	**-2.060**	**.039**
**Total indirect effect of SD on PCL-C via RS**	**-.045**	**-.088**	**-.002**	**.022**	**-2.047**	**.041**
Total indirect effect of ST on PCL-C via RS	-.029	-.061	.003	.016	-1.766	.077
**Total indirect effect of HA on PCL-C via RS**	**.082**	**.011**	**.154**	**.036**	**2.270**	**.023**

S.E.: standard error. Bold values are significant.

PCL-C: Posttraumatic checklist—civilian version; RS: Resilience Scale; NS: novelty seeking; HA: harm avoidance; RD: reward dependence; P: persistence; SD: self-directedness; C: cooperativeness; ST: self-transcendence. PHYS: physical QoL; PSYCH: psychological QoL; SOCIAL: social QoL; ENVIR: environment QoL.

As in the multiple regression models, SEM showed that PTSD symptoms and trait resilience were predicted by harm avoidance, self-directedness, and self-transcendence. Again, the relationship between harm avoidance and self-directedness predicting PTSD symptoms and trait resilience was opposite, and self-transcendence was a positive predictor of PTSD symptoms.

## Discussion

This study investigated the relationship between temperament, character, PTSD symptoms, trait resilience, and QoL in individuals exposed to the Kiss nightclub fire. The main findings were as follows: (1) PTSD symptoms and trait resilience, individually, were predicted by harm avoidance, self-directedness, and self-transcendence; (2) overall QoL was predicted by PTSD symptoms, trait resilience, self-directedness, and cooperativeness; (3) PTSD symptoms and trait resilience mediated the effect of personality on QoL; and (4) PTSD symptoms mediate the relationship of trait resilience and QoL. Given the complexity of the relationships between constructs, we will first discuss the value of these traits individually, and then the dynamic of personality in relation to the outcomes.

### Self-directedness

Basically, self-directedness encompasses maturity and refers to self-determination and the ability to control, regulate, and adapt behavior in accordance with chosen goals and values [27]. High levels have been related with high positive affect, low negative affect, life satisfaction, subjective perceptions of health, and perceived social support [42]. High positive affectivity and low negative affectivity were also related to posttraumatic growth in victims of violence [43]. The literature points out that some characteristics found in self-directed people are related to resilience, such as hope, hardiness, and optimism [25,44]. In longitudinal studies, positive emotion-focused coping (i.e., acceptance, positive reframing, and humor), having a purpose in life, active coping, and perceived social support were associated with better recovery from trauma [17,45,46].

Conversely, low scores in self-directedness were consistently associated with PTSD in cross-sectional studies [30,31,47,48]. This was commonly found in personality, mood, substance dependence, and psychotic disorders, and indicates poor impulse control or weak ego strength [49]. A longitudinal study found a temporal relation between these constructs, showing that low self-directedness before deployment was associated with post-deployment PTSD in a military sample [50].

Self-directedness has been considered as the most important character trait attributed to well-being, life satisfaction, and QoL [51–53]. Studies have found a positive correlation with QoL in clinical patients with schizophrenia and schizoaffective disorder [54], attention deficit and hyperactivity disorder [55], and in samples of psychiatric outpatients [53] and geriatric inpatients [56]. In our study, it was specifically a predictor of psychological QoL, described as emotional and mental wellness, and environmental QoL, described as external resources and opportunities. Additionally, SEM revealed that self-directedness had a direct effect on overall QoL, mediated by trait resilience and PTSD symptoms. Notwithstanding, the effect size of PTSD on QoL was higher than of self-directedness and cooperativeness, indicating that the severity of the disorder has more impact on well-being than personality.

### Harm avoidance

Individuals with high harm avoidance have been described as cautious, fearful, shy, fatigable, apprehensive, nervous, discouraged, and sensitive to criticism and punishment [27,57]. As a temperament trait, it was originally thought to be based on the neurobiology of the behavioral inhibition system, resulting in sensitivity to threatening situations [58]. Harm avoidance was associated with resting signal interaction of the dorsal raphe with the basal amygdala, ventral hippocampus, insula, nucleus accumbens, and medial prefrontal cortex in healthy adults [59]. A longitudinal study with 10 years of follow-up showed that harm avoidance has high temporal stability in adults [60]. We found that harm avoidance was the personality trait that independently accounted for the highest variance in PTSD severity and trait resilience.

It is noteworthy that harm avoidance was strongly and inversely correlated with self-directedness. Fassino et al. (2013) state that these traits together are “the personality functioning core of mental illness, representing both predisposing traits or illness-related factors” [61]. The literature points out that low self-directedness and high harm avoidance have been related to PTSD, whereas the opposite is true for trait resilience [20,21,30,31,48,62]. In fact, this combination was also found in many other psychiatric diagnoses, including substance use disorder, schizophrenia, mood disorders, panic disorder, social phobia, obsessive-compulsive disorder, and eating disorders [61]. Low self-directedness and high harm avoidance were strongly correlated with neuroticism (negative affectivity), a Five-Factor Model trait also highly related to mental disorders [53,63–67]. Additionally, harm avoidance and neuroticism overlap with trait anxiety, which is present in all anxiety disorders, obsessive-compulsive disorder [68], and PTSD [25]. Likewise, in a sample of individuals with rectal cancer, trait anxiety was related to PTSD symptoms and poor QoL [69]. In our study, although harm avoidance was correlated with all dimensions of QoL, the multiple regression showed that, in general, QoL domains were better explained by PTSD severity. Accordingly, SEM evidenced that the effect of harm avoidance on QoL was indirect only, via PTSD and resilience.

### Self-transcendence

Self-transcendent individuals have been described as self-forgetful, creative, altruistic, idealist, transpersonal, and spiritual, whereas those with low self-transcendence as self-striving, individualistic, materialistic, and non-religious [27]. This trait may also be associated with magical thinking and the belief in something abstract, such as an ideology or religion. In our study, self-transcendence was an independent and positive predictor of PTSD severity, a finding that is in accordance with other studies [30,31,47,70]. Facing trauma, death, and suffering arouses thoughts about life and death, good and evil, and justice and wickedness, so that individuals may attribute different meanings to the experience. Individuals with PTSD may have misperceptions regarding their self and surrounds, which include distorted beliefs and dissociation (i.e., depersonalization and a feeling of unreality) [71]. A study on Oakland/Berkeley firestorm survivors found that self-transcendence was specifically associated with avoidance/numbing PTSD symptoms [48]. Additionally, religious coping, described as a primary means of dealing with suffering by seeking solace in religion, was associated with a worse PTSD trajectory in a 12-year follow-up study with World Trade Center responders [45].

Interestingly, self-transcendence was also a positive predictor of trait resilience, subjective QoL, and spiritual QoL. As far as we know, only two studies found that trait resilience was predicted by self-transcendence (using TCI), whereas other research did not confirm these findings. An investigation of musculoskeletal pain patients found that self-transcendence was a positive predictor of many aspects of well-being, including resilience, life satisfaction, positive affect, energy, and harmony in life [72]. Kim, Lee, and Lee (2013), in a large sample of students, showed that self-transcendence was a positive predictor of resilience [21]. A longitudinal study showed that altruism, an aspect of self-transcendence, was associated with resilience in veterans [17] and positive emotions in community volunteers [42]. Self-transcendence and self-directedness were predictors of spiritual QoL in depressed outpatients [73]. Our study points out that some self-transcendent people may see their QoL as satisfactory and be simultaneously symptomatic and resilient [74]. It may be attributed to spirituality, religiousness, and personal beliefs, which should attribute a positive meaning even to bad experiences.

Self-transcendence has been related to mental health and mental disorders, and may be better understood in conjunct with other character traits [42,60]. Together with low self-directedness and cooperativeness, high self-transcendence is related to a tendency for dissociative and psychotic symptoms [75–78]. In contrast, in individuals with high self-directedness and cooperativeness, high self-transcendence indicates maturity, spirituality, and creativity, and has been associated with positive affect and life satisfaction [42,51,75].

### Cooperativeness

Cooperativeness was strongly correlated with self-directedness, moderately correlated with PTSD symptoms, and weakly with resilience. This trait refers to the interpersonal domain of personality. Individuals with low cooperativeness are described as being intolerant, narcissistic, hostile or disagreeable, revengeful, and opportunistic [79]. In a study by North, Abbacchi, and Cloninger (2012) on survivors of the Oklahoma City bombing, the combination of low cooperativeness and low self-directedness doubled the probability of PTSD [31]. Low cooperativeness was also related to suicidal ideation in PTSD patients, adding a severity component to the evaluation of these cases [80]. Furthermore, low scores for self-directedness and cooperativeness strongly indicate personality disorders, whereas high scores on these two measures indicate maturity [75,79,81–84]. Both traits were related to overall QoL in SEM model, which indicates that ego maturity is associated to a better QoL in traumatized patients. However, the finding that cooperativeness presented a positive relationship with PTSD symptoms in SEM was quite surprising, as literature suggests to the opposite. It raises the questioning if characteristics of cooperativeness would be associated with vulnerability to PTSD. This issue must to be explored in further studies.

### Other traits

Other personality traits, such as novelty seeking and reward dependence, were not correlated with PTSD symptoms or with resilience. High novelty seeking, high harm avoidance, and low reward dependence, as measured by the Tridimensional Personality Questionnaire, were associated with PTSD symptoms in two studies on combat-related PTSD [85,86]. Evren et al. (2010) identified that alcohol-dependent inpatients with PTSD have higher mean scores for novelty seeking, harm avoidance, and self-transcendence than those without PTSD, but in a multivariable model, only self-directedness and age predicted PTSD [47].

### Limitations

The main limitation of this study concerns the inference of causality. First, a temporal relation, which would suggest causality, would be better evaluated with longitudinal measurements. Nevertheless, a study with undergraduate students exposed to a terrorist explosion found that the risk of developing PTSD within six months was positively associated with harm avoidance and negatively with novelty-seeking, measured prior to the exposure [87]. There is also some evidence from longitudinal studies that self-directedness and harm avoidance indicate vulnerability to depression [26,88,89]. Moreover, the assessment of personality traits in clinical samples may be influenced by the emotional state of symptomatic individuals, which might interfere with questionnaire answers. For example, depression severity is measured based on harm avoidance and self-directedness, but these may change over time with psychotherapy or antidepressants [90]. Therefore, the findings should not be interpreted as meaning that the personality traits have a causal relationship in the analyzed outcomes, but rather that individual characteristics are more likely to be found in individuals with more or less PTSD severity, trait resilience, or QoL in the context of a man-made disaster.

Other possible limitation is that the study used two subsamples, of victims and professional first responders. As it would represent different profiles of personality, measures of personality, resilience and PTSD should be biased by subsamples. To reduce the possibility of bias, we conducted all analyses controlling for type of exposure, sex differences and current psychiatric treatment. Further studies may conduct separate analysis for both groups in order to explore personality characteristics in this two groups of individuals exposed to Kiss nightclub fire.

## Conclusion

This study confirmed our hypothesis that temperament and character traits would be related to PTSD symptoms, trait resilience, and QoL in a sample of people exposed to the Kiss nightclub fire. Self-directedness was a positive and independent predictor of trait resilience and QoL. Low self-directedness was related to PTSD symptoms and worse QoL. Of the personality traits, harm avoidance was the strongest positive predictor of PTSD symptoms and a negative predictor of trait resilience and subjective QoL. Self-transcendence was positively related to PTSD symptoms, trait resilience, subjective QoL, and spiritual QoL; thus, this trait may indicate a coping style that may coexist with psychopathology. Moreover, compared to other personality traits, self-directedness was the strongest predictor of total QoL, with a direct effect and indirect effects that were mediated by PTSD symptoms and resilience. In other words, ego strength, with self-determining behavior, reflects directly on QoL, but this effect is under the influence of posttraumatic psychopathology and the ability to cope.

These findings may contribute to clinical investigations of pathological patterns of personality related to PTSD symptoms, identify strengths and adaptive coping measures, and plan specific interventions to improve QoL. Lastly, we encourage longitudinal studies to explore the causal relationship between personality and psychopathology.
